# Network-based identification of microRNAs as potential pharmacogenomic biomarkers for anticancer drugs

**DOI:** 10.18632/oncotarget.10052

**Published:** 2016-06-14

**Authors:** Jie Li, Kecheng Lei, Zengrui Wu, Weihua Li, Guixia Liu, Jianwen Liu, Feixiong Cheng, Yun Tang

**Affiliations:** ^1^ Shanghai Key Laboratory of New Drug Design, School of Pharmacy, East China University of Science and Technology, Shanghai, China; ^2^ State Key Laboratory of Biotherapy/Collaborative Innovation Center for Biotherapy, West China Hospital, West China Medical School, Sichuan University, Chengdu, China; ^3^ Current address: Center for Cancer Systems Biology, Dana-Farber Cancer Institute, Harvard Medical School, Boston, USA; ^4^ Current address: Center for Complex Networks Research, Northeastern University, Boston, USA

**Keywords:** pharmacogenomics, miRNA, network-based inference, metformin, breast cancer

## Abstract

As the recent development of high-throughput technologies in cancer pharmacogenomics, there is an urgent need to develop new computational approaches for comprehensive identification of new pharmacogenomic biomarkers, such as microRNAs (miRNAs). In this study, a network-based framework, namely the SMiR-NBI model, was developed to prioritize miRNAs as potential biomarkers characterizing treatment responses of anticancer drugs on the basis of a heterogeneous network connecting drugs, miRNAs and genes. A high area under the receiver operating characteristic curve of 0.820 ± 0.013 was yielded during 10-fold cross validation. In addition, high performance was further validated in identifying new anticancer mechanism-of-action for natural products and non-steroidal anti-inflammatory drugs. Finally, the newly predicted miRNAs for tamoxifen and metformin were experimentally validated in MCF-7 and MDA-MB-231 breast cancer cell lines via qRT-PCR assays. High success rates of 60% and 65% were yielded for tamoxifen and metformin, respectively. Specifically, 11 oncomiRNAs (e.g. miR-20a-5p, miR-27a-3p, miR-29a-3p, and miR-146a-5p) from the top 20 predicted miRNAs were experimentally verified as new pharmacogenomic biomarkers for metformin in MCF-7 or MDA-MB-231 cell lines. In summary, the SMiR-NBI model would provide a powerful tool to identify potential pharmacogenomic biomarkers characterized by miRNAs in the emerging field of precision cancer medicine, which is available at http://lmmd.ecust.edu.cn/database/smir-nbi/.

## INTRODUCTION

**Figure 1:**
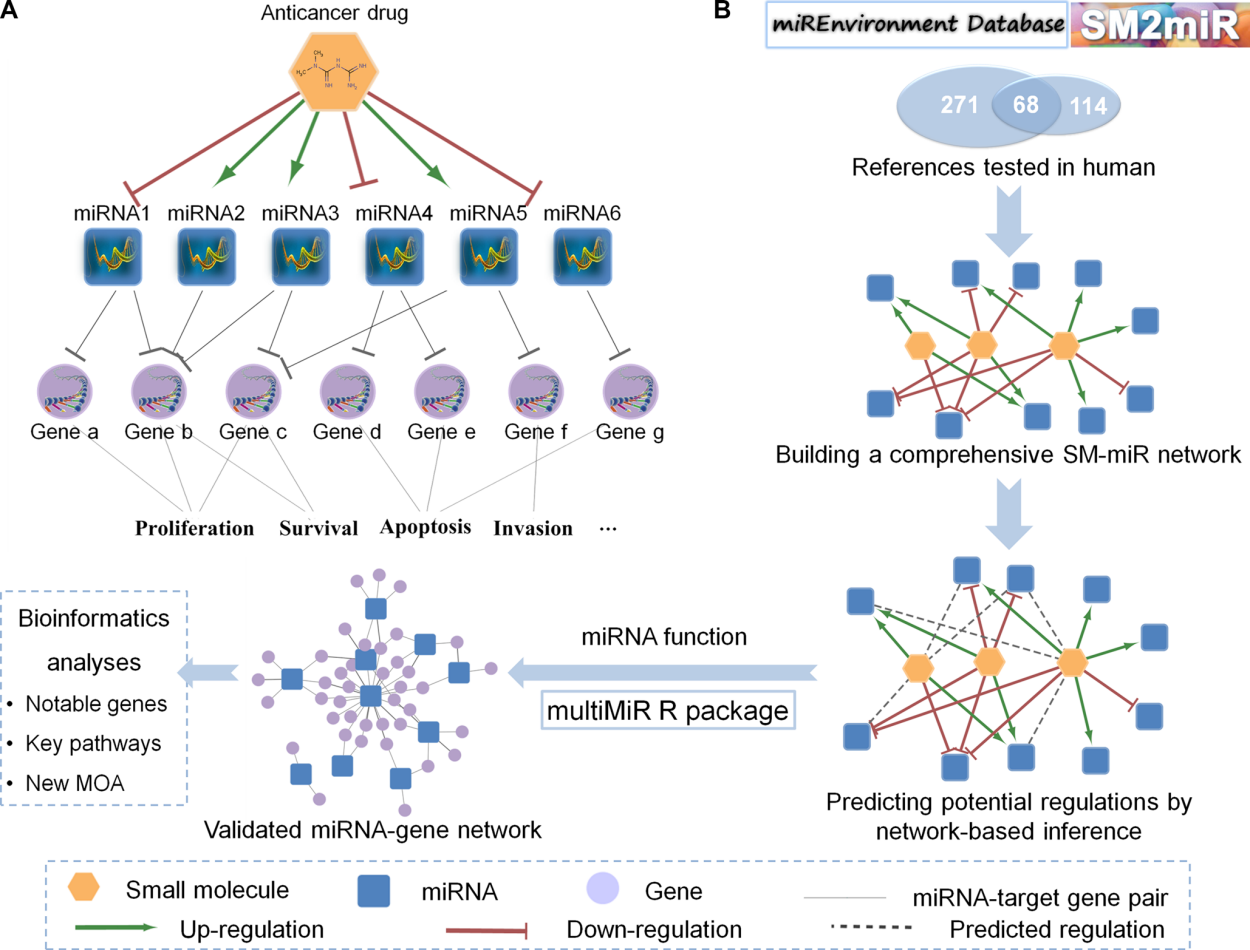
General diagrams of the SMiR-NBI model for miRNA-mediating cancer pharmacogenomic studies (**A**) The biological diagram of miRNA pharmacogenomics. MiRNAs can be up-/down-regulated by an anticancer drug, then directly target the downstream genes to mediate its anticancer responses. (**B**) The workflow of miRNA pharmacogenomics. The predictive Small Molecule-miRNA Network-Based Inference (SMiR-NBI) model was built by network-based inference algorithm, based on the curated heterogeneous network connecting small molecules and miRNAs (SM-miR network). The miRNA-mediating mechanism-of-action (MOA) of anticancer responses was annotated by bioinformatics analyses on the miRNA-gene function network.

Genetic profiles or molecular features often characterize the responses (e.g. resistance) of an individual to anticancer treatment [1]. Identification of those genetic profiles or molecular features will help to find the right drug with right dosage for the right person in the era of precision medicine [2]. The traditional pharmacogenomic studies focus on identifying which drugs will be the most effective with low toxicity for a particular patient harboring unique genetic profiles, such as single nucleotide polymorphisms, somatic copy number alterations or differential expressions of drug targets, drug-metabolizing enzymes or transporters [3]. Recent studies suggest that microRNAs (miRNAs) might play critical roles in pharmacogenomics [4, 5]. MiRNA pharmacogenomics is to study treatment responses at the miRNA level (Figure 1A): to decrease or increase the expressions of miRNAs, target genes or to change the activities of binding miRNAs [6]. In the context of cancer pharmacogenomics, miRNA biomarkers have been found to be involved in intrinsic and acquired resistance to cancer therapies by decreasing the expressions of their target genes [4].

Compared with other miRNA regulation strategies, like locked nucleic acids (LNA), small molecules attracted increasing attention for its strength in organism affinity and extensive experience in clinical research and pharmacokinetic test [7]. MiRNA regulation by small molecules or drugs could result from inference in miRNA biogenesis at three levels: before, during and after transcription [7]. Small molecules increase or decrease miRNA expressions indirectly, by altering miRNA promoter regions [8] or binding to the transcription factors [9]. They also can disrupt the maturation of miRNAs by binding with essential RNA-endonucleases [10]. In general, one small molecule can alter tens of miRNAs, and one miRNA can target ∼200 genes in average [11]. For example, a total of 132 miRNAs were altered in an array analyses of HCT116 colon cancer cells treated by sulindac sulfide [12]. However, it is time-consuming to identify the regulations between small molecules and miRNAs by experimental approaches owing to the high complexity of biological systems. Therefore, there is an urgent need to develop new computational approaches or models to systematically decipher the relationships between anticancer agents and their treatment responses mediated by miRNAs to speed up cancer pharmacogenomic studies [13].

As the advance of high-throughput technologies, miRNA-related online resources sprung up, mainly focusing on miRNA identification, target gene validation/prediction, and function annotation [14, 15]. MiRBase [16], the reference database for miRNA annotation, has enrolled 2,588 human mature miRNAs in the latest release (Version 21, June 2014). The miREnvironment [17] and SM2miR [18] databases are created by text-mining, including literature-curated associations between xenobiotics and miRNAs. In addition, several miRNA target gene databases, such as miRTarBase [11], TarBase [19] and miRecords [20] curated thousands of the target genes for miRNAs supported by experimental data. The multiMiR [21] is an integrated R package, including various resources of the validated and predicted miRNA-target gene pairs. Those databases provide high-quality data for the development of new computational models for miRNA pharmacogenomic studies, such as network-based approaches [4]. Lv *et al*. built a network-based computational model to predict novel regulations between small molecules and miRNAs on the basis of the integrated similarities using Random Walk with Restart algorithm [22]. Meng *et al*. constructed a rank-based model to predict potential modulators for 25 cancer significant miRNAs based on gene expression similarity [23]. Currently, there is still a great need for feasible, high efficient and/or accuracy models for comprehensive evaluation of the regulations between small molecules and miRNAs in large-scale.

In this study, a network-based miRNA pharmacogenomic framework, namely the predictive Small Molecule-miRNA Network-Based Inference (SMiR-NBI) model, was developed to discover the underlying mechanisms of anticancer drug responses mediated by miRNAs. The SMiR-NBI model was built based on a heterogeneous network connecting drugs, miRNAs and genes, using our previously developed network-based inference (NBI) framework [24]. The SMiR-NBI model, with high accuracy and low computational cost, only utilized the network topology information from the constructed heterogeneous network as input [25]. Further, network and bioinformatics analyses were used to identify miRNAs as potential pharmacogenomic biomarkers in cancer. Altogether, our SMiR-NBI model displayed high performance in cross-validation and experimental validation in several case studies, and would provide a valuable computational tool for miRNA pharmacogenomic studies in the emerging field of precision cancer medicine.

## RESULTS

### Drug-miRNA network building and topological analyses

A comprehensive network connecting small molecules and miRNAs (abbreviated as SM-miR network) was built based on the data collected from the miREnvironment [17] and SM2miR [18] databases. Only experimental records from 453 references tested in human were used. After removing duplicates and performing data standardization, the final high-quality dataset included 2,447 SM-miR regulations connecting 154 small molecules and 618 human mature miRNAs (Table 1). Among them, 1,359 regulations were labeled as up-regulation, while 1,088 regulations were annotated as down-regulation. In order to illustrate the function of the regulated miRNAs, the genes directly targeted by those miRNAs were extracted using the multiMiR R package [21]. For 618 miRNAs in the SM-miR network, 376 ones were recorded with validated target genes. In total, 3,025 miRNA-target gene pairs supported by strong validations and 32,648 miRNA-target gene pairs supported by weak validations were obtained.

**Table 1:** Statistics of the heterogeneous network connecting small molecules, miRNAs and target genes

Network Name	Type	SM^c^	miRNAs	Genes	Associations
SM-miR net^a^	Up-regulation	132	503	/	1,359
	Down-regulation	100	377	/	1,088
	Total	154	618	/	2,447
miRNA-gene net^b^	Strong validation	/	288	1,442	3,025
	Weak validation	/	282	11,820	32,648
	Total	/	376	12,085	35,673

a SM-miR net: the network of associations connecting small molecules and miRNAs;

b miRNA-gene net: the network of associations connecting miRNAs and their target genes;

c SM: small molecules.

**Figure 2:**
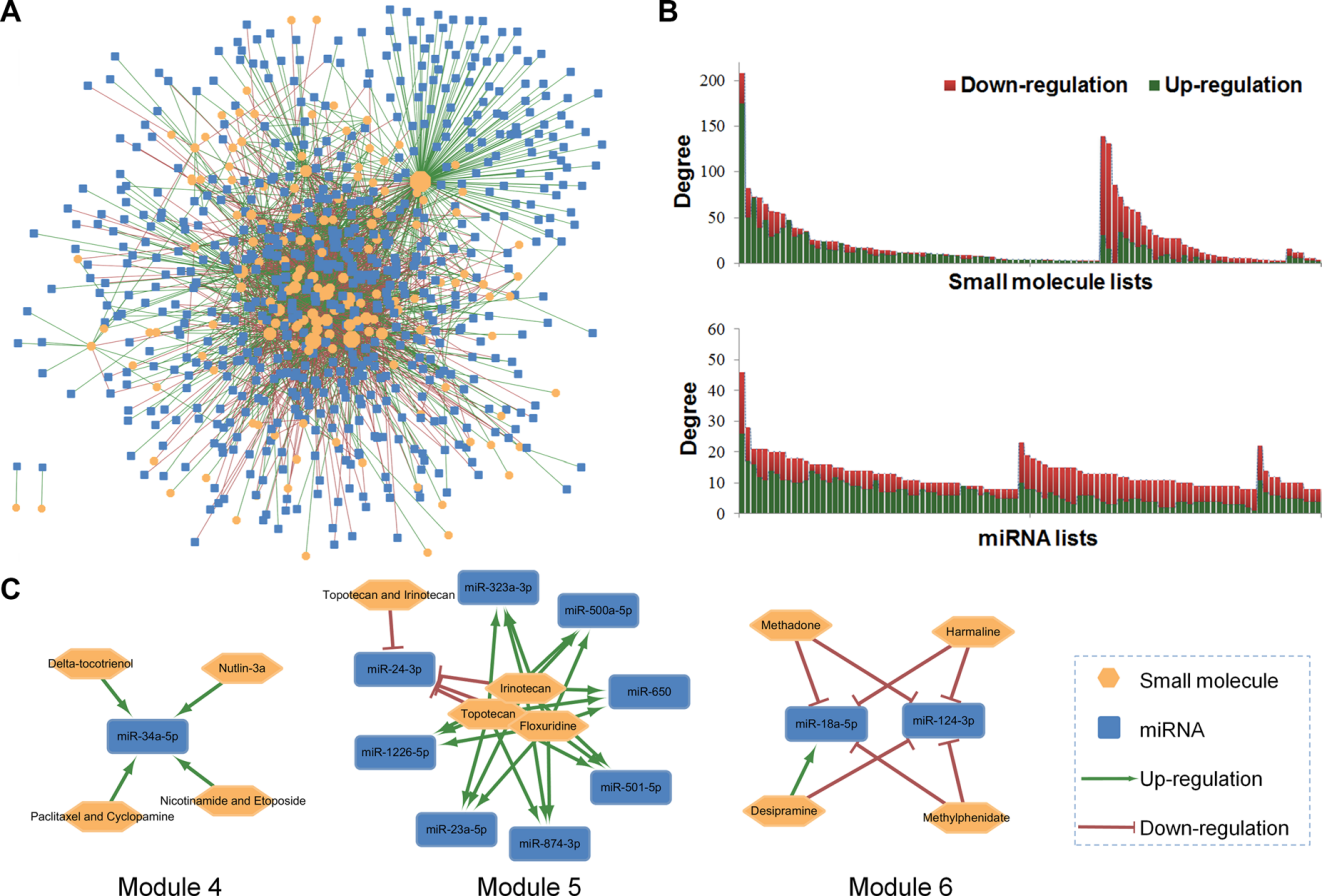
Building a SM-miR network connecting small molecules (SM) and miRNAs and network topological analyses (**A**) Global diagram of the known SM-miR network containing 2,447 up-/down-regulations between 154 small molecules and 618 miRNAs; (**B**) The degree distribution for the top 100 small molecules and miRNAs; (**C**) Module 4-6, with clustered small molecules and their co-regulated miRNAs.

The global SM-miR network diagram (Figure 2A) was constructed by Cytoscape [26], including 2,447 SM-miR regulations. Most of the included drugs were connected each other by the co-regulated miRNAs, except the isolated benzothiazole and lovastatin owing to data incompleteness. To measure the topological features, degree (K) [27] was calculated by “Network analyzer” tool in Cytoscape. The statistics results for the nodes with the top 100 global degrees were displayed in Figure 2B. MiR-21-5p with the highest degree (K = 47) was the most studied miRNA, which was up-/down-regulated by 47 small molecules. Several miRNAs, including let-7a-5p, miR-16-5p, miR-27b-3p, miR-29a-3p, miR-34a-5p, miR-125b-5p, and miR-181b-5p, were up-/down-regulated by more than 20 small molecules or drug combinations. Three drugs with the highest degrees, namely 5-fluorouracil, 1,2,6-tri-o-galloyl-beta-d-glucopyranose and sulindac sulfide, regulated the expression levels of 208, 139 and 131 miRNAs, respectively. Although multiple miRNAs were up-/down-regulated by multiple drugs, specificity for up-/down-regulation still existed. For instance, the drug combination of 5-aza-2′-deoxycytidine and trichostatin A increased the expression levels of all 47 linked miRNAs.

We further investigated the network modules (Supplementary Figure S1) on the global SM-miR network using the MCODE algorithm [28]. Drugs clustered in a module often share the same mechanism-of-action (MOA), with commonly regulated miRNAs displayed in Module 4-6 (Figure 2C). For example, miR-34a-5p may act as a common biomarker for 4 therapeutic strategies (Module 4): delta-tocotrienol, nutlin-3a, the drug combination of nicotinamide and etoposide, and the drug combination of paclitaxel and cyclopamine. The difference in topological features defined the various initial resources for small molecules or miRNAs, providing the basis to conduct network-based prediction.

### Identification of new miRNAs as cancer pharmacogenomic biomarkers by the SMiR-NBI model

A miRNA pharmacogenomic framework (Figure 1B) was built to discover potential miRNA biomarkers characterizing the responses of anticancer drugs via three steps (see Methods): 1) constructing the high-quality reported heterogeneous network connecting small molecules and their regulated miRNAs; 2) predicting novel SM-miR regulations using network-based inference algorithm [24]; 3) investigating the miRNA-mediating MOA by bioinformatics analyses based on the miRNA-gene function network.

**Figure 3:**
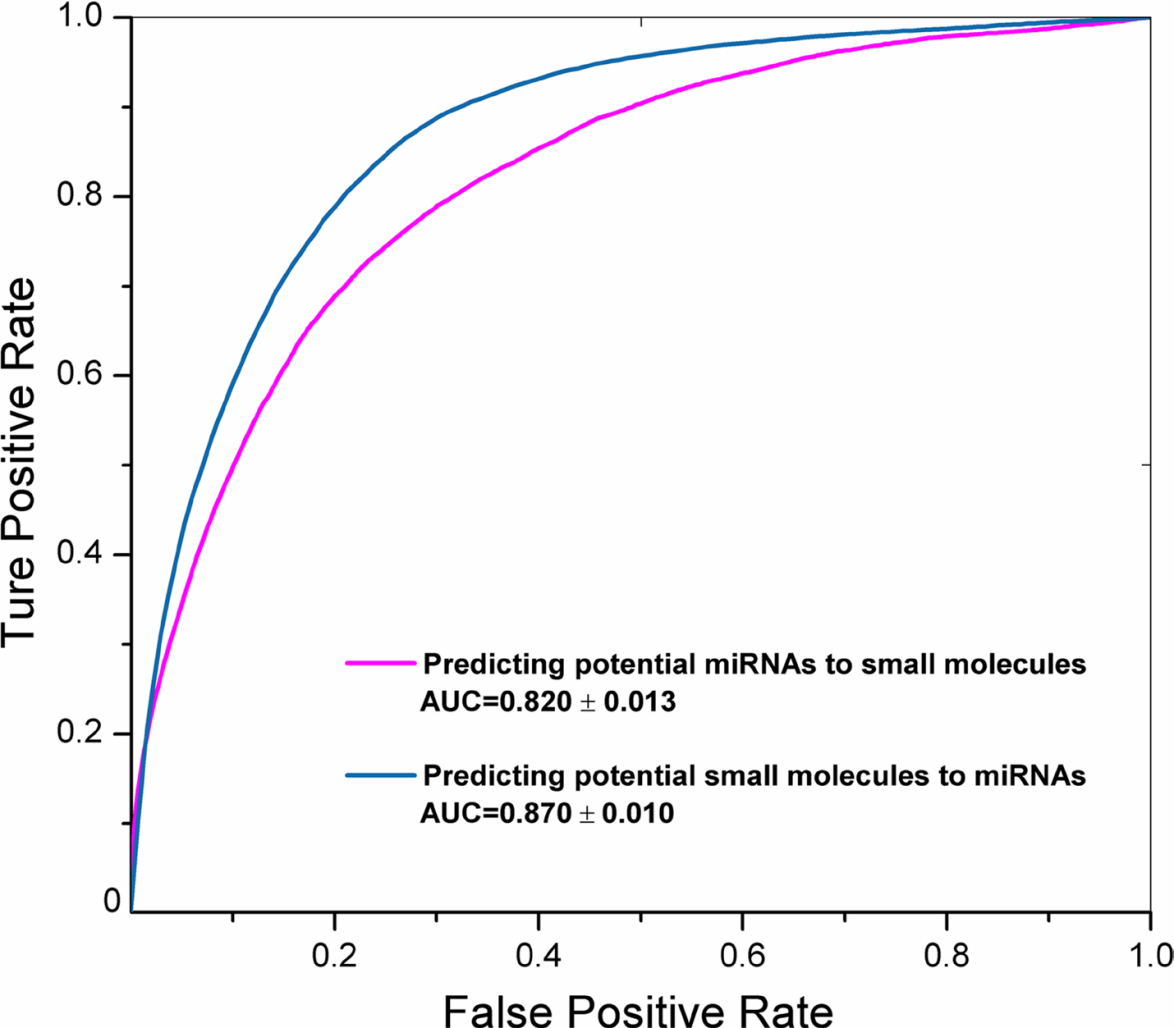
The receiver operating characteristic (ROC) curves of the SMiR-NBI model The areas under the ROC curves (AUC) were labeled with means and standard errors using 100 times of 10-fold cross validation for predicting potential miRNAs to a given small molecule (magenta line) or predicting potential small molecules to a miRNAs of interest (steel blue line) via our previously developed network-based inference framework [24].

The SMiR-NBI model can rank new miRNAs for a given small molecule or predict new small molecules for a miRNA of interest via its personalized network-based inference as described in our previous study [24, 25]. The performance of the SMiR-NBI model was measured by the areas under the receiver operating characteristic curves (AUC) during 100 times of 10-fold cross validation (Figure 3). A high AUC value of 0.820 ± 0.013 was yielded for predicting potential miRNAs to small molecules. In addition, a higher AUC value of 0.870 ± 0.010 was yielded for predicting potential small molecules to miRNAs. In total, 5,245 novel SM-miR regulations (score ≥ 0.1) were identified by the SMiR-NBI model for all included small molecules that we studied, except benzothiazole and lovastatin owing to lack the topological links of those two molecules with the whole network (Figure 2A). We integrated the predicted list with the previously reported SM-miR network collected from the miREnvironment [17] and SM2miR [18] databases with miRNA target genes extracted using the multiMiR R package [21]. All known and computationally predicted SM-miR regulations and miRNA function information were available on our website (http://lmmd.ecust.edu.cn/database/smir-nbi/) for the future experimental validations.

**Figure 4:**
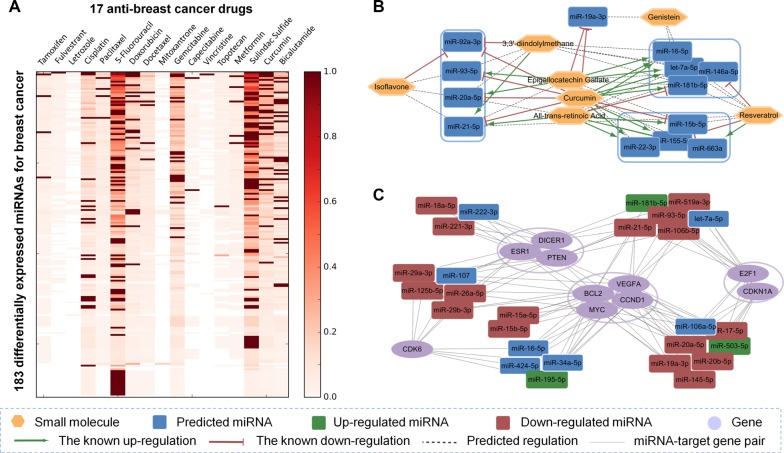
Identification of potential pharmacogenomic biomarkers for breast cancer, natural products and non-steroidal anti-inflammatory drugs (NSAIDs) via the SMiR-NBI model (**A**) The heatmap showed the predicted scores (color keys) of the SMiR-NBI model for 183 differentially expressed miRNAs in breast cancer from The Cancer Genome Atlas (see Methods) and 17 anti-breast cancer drugs. (**B**) The representative miRNA pharmacogenomic pathway for natural products included 13 common miRNAs regulated by at least 3 different natural products. (**C**) The representative miRNA pharmacogenomic pathway for NSAIDs contained 10 hub genes with the highest degrees in NSAIDs-regulating miRNA-gene subnetwork and 27 miRNAs targeting at least 3 hub genes. Regulations between NSAIDs and miRNAs were denoted by different colors.

To further examine the performance of our SMiR-NBI model, we calculated the differentially expressed miRNAs in breast invasive carcinoma (BRCA) from The Cancer Genome Atlas (TCGA) [29] as a case study. Totally, 265 miRNAs were identified as differentially expressed miRNAs in BRCA using a cut-off: |log_2_(fold change [FC])| > 1 and adjusted *p*-value < 0.05. Among the 265 differentially expressed miRNAs, 183 mature miRNAs were predicted to be involved in the treatment responses to 17 anti-breast cancer small molecules via the SMiR-NBI model (Supplementary Table S1 and Figure 4A), displaying the reliability of prediction by the SMiR-NBI model. On the other aspect, to illustrate the real application ability of the SMiR-NBI model for prioritizing potential miRNA biomarkers to small molecules, we further exemplified natural products and non-steroidal anti-inflammatory drugs (NSAIDs) as two case studies.

### Discovery of new miRNAs mediating anticancer responses for natural products

Contrasted with other small molecules, natural products have their immanent advantages like human body friendly and low toxicity properties. Recently, anticancer mechanisms for natural products have drawn great attention, especially those mediated by the critical miRNA pharmacogenomic biomarkers [30, 31]. We systematically studied the miRNA pharmacogenomic profiles for 7 natural small molecules included in the SMiR-NBI model (Supplementary Table S2). The whole miRNA pharmacogenomic profiles for natural products contained the known up-/down-regulated miRNAs previously reported in the published literature (shown in green or red, respectively), the novel SM-miR regulations predicted by the SMiR-NBI model (shown in gray), and the hub target genes for the miRNAs. In total, 82 miRNAs were previously reported or computationally predicted as biomarkers for curcumin, with 40 miRNAs for epigallocatechin gallate (EGCG), 37 miRNAs for all-trans-retinoic acid (ATRA), 13 miRNAs for isoflavone and resveratrol, and 12 miRNAs for genistein and 3,3′-diindolylmethane. The common miRNAs regulated by at least three different natural products resulted in 13 co-regulated miRNAs, including let-7a-5p, miR-16-5p, and miR-21-5p (Figure 4B). These miRNAs may play crucial roles by mediating treatment responses for natural products.

The downstream gene network for natural product-mediating miRNA pharmacogenomics was next examined by bioinformatics analyses to illustrate molecular mechanisms. As shown in Supplementary Table S2, two cancer genes, namely the *BCL2* (B-cell CLL/lymphoma 2) and the *PTEN* (Phosphatase and tensin homolog), were hub nodes in bioinformatics analyses. *BCL2* was found to be targeted by 39 miRNAs, and *PTEN* was directly targeted by 28 miRNAs including oncogenic miR-17-92 family (extracted from our collected miRNA-target gene network). The SMiR-NBI model predicted some novel SM-miR regulations for natural products in the *BCL2* pathway and the *PTEN* pathway. For instance, miR-16-5p, an endogenous antisense to treat *BCL2*-overexpressing tumors [32], was predicted by the SMiR-NBI model as a potential biomarker to anticancer responses for resveratrol, genistein and 3,3′-diindolylmethane (Figure 4B). In addition, miR-19a-3p, a member of miR-17-92 family involving in *PTEN* anticancer pathway, was predicted as a potential biomarker for genistein via the SMiR-NBI model (Figure 4B). Altogether, the predicted lists via the SMiR-NBI model would provide potential pharmacogenomic biomarkers for understanding the treatment responses of natural products.

### Discovery of new miRNAs characterizing anticancer indications by NSAIDs

The MOA for the anticancer indications of non-steroidal anti-inflammatory drugs (NSAIDs) is poorly understood [33]. Herein, a comprehensive miRNA pharmacogenomic subnetwork for NSAIDs (Supplementary Figure S2) was investigated to search potential miRNAs mediating treatment responses for two classic NSAIDs: sulindac sulfide and celecoxib. Supplementary Figure S2 displayed 2,377 miRNA-target genes pairs with strong experimental evidence for 182 both previously reported or computationally predicted miRNAs via the SMiR-NBI model.

Among the miRNA pharmacogenomic subnetwork for NSAIDs (Supplementary Figure S2), inflammation, as one of the cancer hallmarks [34], was displayed a hub. Thus, several inflammation-related genes (i.e. *PTGS2*, *IL11*, *IL13*, *IFNG*, *STAT3* and *NFKB1*) were extracted from the whole miRNA-target gene network (Supplementary Table S3). We found that several new predicted miRNAs for NSAIDs via the SMiR-NBI model may mediate anti-inflammatory pathways. For example, 6 miRNAs were predicted as potential biomarkers for COX-2-characterizing pathway (COX-2 encoded by *PTGS2*), with 4 potential miRNAs for interleukin, and 3 potential miRNAs for *STAT3*. In addition, among several miRNA-mediating key inflammatory factors, NF-κB was well identified in the previous study [12]. Finally, miR-16-5p displayed a potential anti-inflammatory biomarker of NSAIDs responses for future experimental validations, with high predicted scores and directly targeting *PTGS2*, *IFNG* and *NFKB1*.

In addition to the inflammation pathway, several cancer-related pathways were identified from the global NSAIDs-regulating miRNA-gene subnetwork, including cell cycle (*CDK6*, *CCND1*, *CCKN1A*, and *E2F1*), proliferation (*MYC*), apoptosis (*BCL2*) and angiogenesis (*VEGFA*) [35]. Figure 4C showed a representative miRNA pharmacogenomic subnetwork for NSAIDs connecting the 10 hub genes (with the highest degrees) and 27 miRNAs (targeting at least 3 hub genes). Among 50 newly predicted miRNAs for NSAIDs, 7 miRNAs with high degree distribution revealed potential candidates for future experimental validations. For example, the predicted miR-222-3p was clustered with down-regulated miR-221-3p and miR-18a-5p by directly targeting *DICER1*, *ESR1* and *PTEN*. The newly predicted miR-107 shared the similar functional pathways with down-regulated miR-29 family. Collectively, the newly the top 10 predicted miRNAs (Figure 4C) via the SMiR-NBI model would provide potential miRNA pharmacogenomic candidates mediating treatment anticancer responses for NSAIDs, although future efforts are needed to perform experimental validations.

### Experimental validation of new miRNAs characterizing tamoxifen responses in MCF-7 breast cancer cells

**Figure 5:**
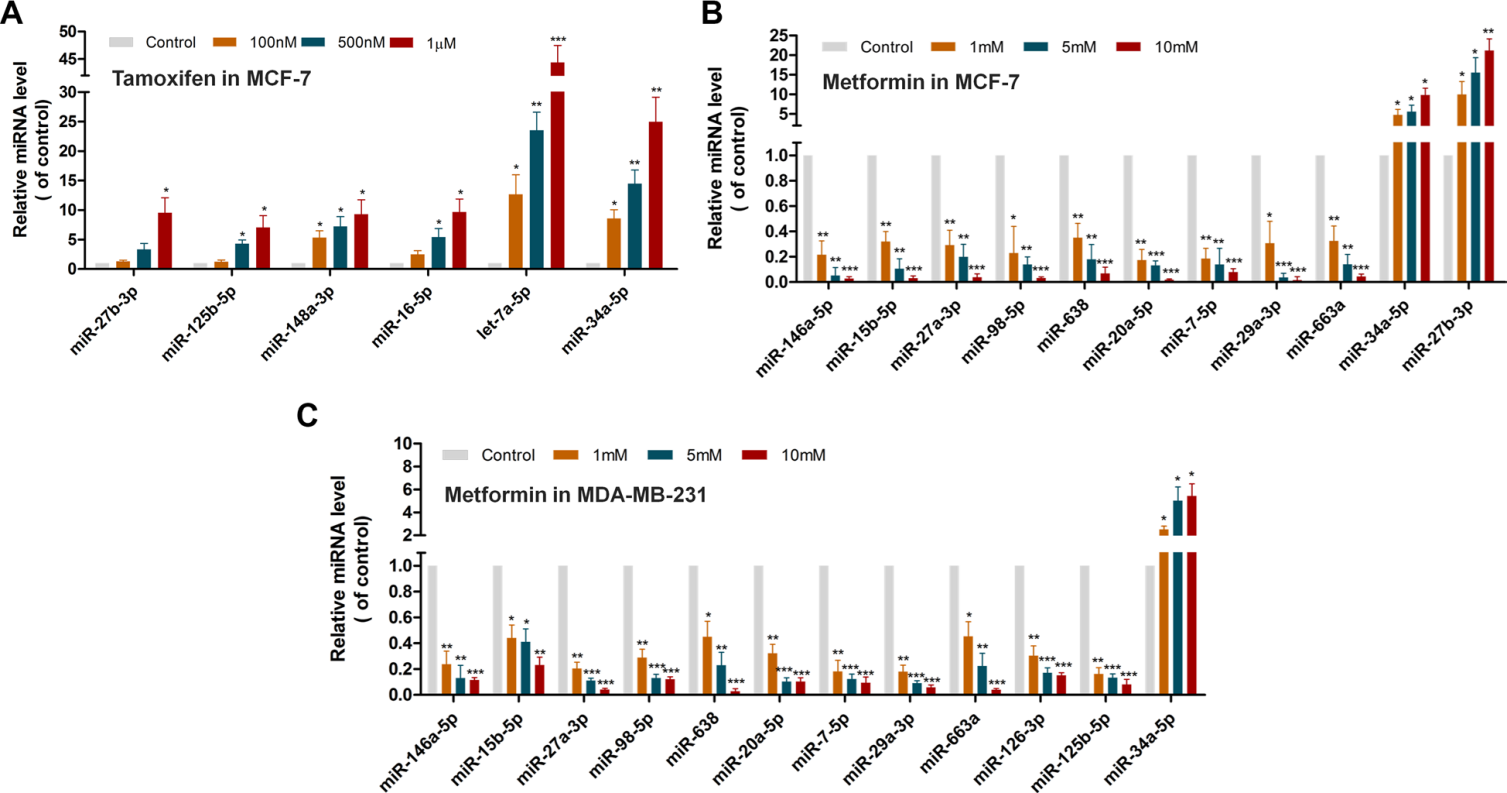
Discovery of new miRNAs mediating treatment responses for tamoxifen and metformin in MCF-7 or MDA-MB-231 cell lines via qRT-PCR assays (**A**) The qRT-PCR assay for expression change of 6 predicted miRNAs among the top 10 predicted candidates in the MCF-7 cells treated with 100 nM, 500 nM or 1 μM tamoxifen respectively. (**B**) The qRT-PCR assay for expression change for 11 predicted miRNAs among the top 20 predicted candidates in the MCF-7 cells treated with 1 mM, 5 mM or 10 mM metformin respectively. (**C**) The qRT-PCR assay for expression change of 12 predicted miRNAs among the top 20 predicted candidates in the MDA-MB-231 cells treated with 1 mM, 5 mM or 10 mM metformin respectively. *p < 0.05, **p < 0.01 and ***p < 0.001 were determined by *t*-test. Error bars represent standard errors (s.d., *n* = 3).

Recent studies have suggested that miRNAs may play important roles in anti-breast cancer effects and drug responses [36, 37]. In this study, we predicted new miRNAs for tamoxifen via the SMiR-NBI model and tested prediction experimentally through the quantitative reverse transcription-PCR (qRT-PCR) assays. For tamoxifen, the top 10 predicted miRNAs with the highest scores by the SMiR-NBI model were selected to test using the qRT-PCR assays in two breast cancer cell lines. Fold-change was calculated after the tamoxifen treatment (100 nM, 500 nM and 1 μM, respectively) with the normalized miRNA expression in the control sample without tamoxifen. In total, 6 miRNAs (let-7a-5p, miR-16-5p, miR-27b-3p, miR-34a-5p, miR-125b-5p and miR-148a-3p) revealed the elevated expression levels with fold-change > 2 by a dose-dependent manner in a hormone-positive (both estrogen and progesterone receptors) breast cancer line, MCF-7 (Figure 5A). It indicated a 60% success rate for predicted miRNAs to tamoxifen via the SMiR-NBI model validated in MCF-7 cells. Among 6 miRNAs, let-7a-5p was the most up-regulated one with over 10-fold changes caused by tamoxifen in all three different concentrations, consistent with a previous study [38]. We next tested the top 10 potential miRNAs in a triple negative breast cancer (TNBC) cell line, MDA-MB-231, using the qRT-PCR assays. Surprisingly, none showed over 2-fold changes by tamoxifen in a dose-dependent manner in MDA-MB-231 cells. These results indicated that tamoxifen may show strong cell type-specific miRNA pharmacogenomic biomarkers in TNBC versus hormone-positive breast cancer [36].

### Experimental validation of new miRNAs characterizing metformin responses in both MCF-7 and MDA-MB-231 cell lines

Metformin, the most prescribed oral anti-diabetic agent for type II diabetes, has recently been under phase III clinical trials for breast cancer therapies (http://www.clinicaltrials.gov). Among the predicted list for metformin via the SMiR-NBI model, we tested the expression levels for the top 20 predicted miRNAs for metformin via the qRT-PCR assays in both MCF-7 and MDA-MB-231 breast cancer cell lines. Figure 5B showed that 9 miRNAs (miR-7-5p, miR-15b-5p, miR-20a-5p, miR-27a-3p, miR-29a-3p, miR-98-5p, miR-146a-5p, miR-638, and miR-663a) displayed the decreased expression levels with fold-change < 0.5 in MCF-7 cell lines after metformin treatment. In contrast, 2 miRNAs (miR-34a-5p and miR-27b-3p) revealed the elevated expression levels with fold-change > 2 by a dose-dependent manner (Figure 5B). For MDA-MB-231 cells, 11 miRNAs showed the decreased expression with fold-change < 0.5 after metformin treatment, including 2 more miRNAs (miR-125b-5p and miR-126-3p) compared to MCF-7 cells. Smilar to MCF-7 cells, one miRNA (miR-34a-5p) revealed the elevated expression with fold-change > 2 after metformin treatment responses in a dose-dependent manner (Figure 5C). In total, 13 predicted miRNAs for metformin were experimentally validated in the qRT-PCR assays, accounting for 65% success rate. Those newly identified miRNAs may provide potential pharmacogenomic biomarkers for characterizing treatment responses for metformin in breast cancer. For instance, miR-27a-3p, an oncomiRNA and a biomarker for breast cancer progression [39], was firstly validated as a potential biomarker for metformin via the SMiR-NBI model. In addition, two down-regulated miRNAs by metormin, i.e. miR-29a-3p and miR-146a-5p, were associated with treatment of drug-resistant breast cancer [40, 41]. The 10 newly identified miRNAs for metformin were overlapped between MCF-7 and MDA-MB-231 cell lines, suggesting the non-cell type-specific pharmacogenomic biomarkers for metformin.

**Figure 6:**
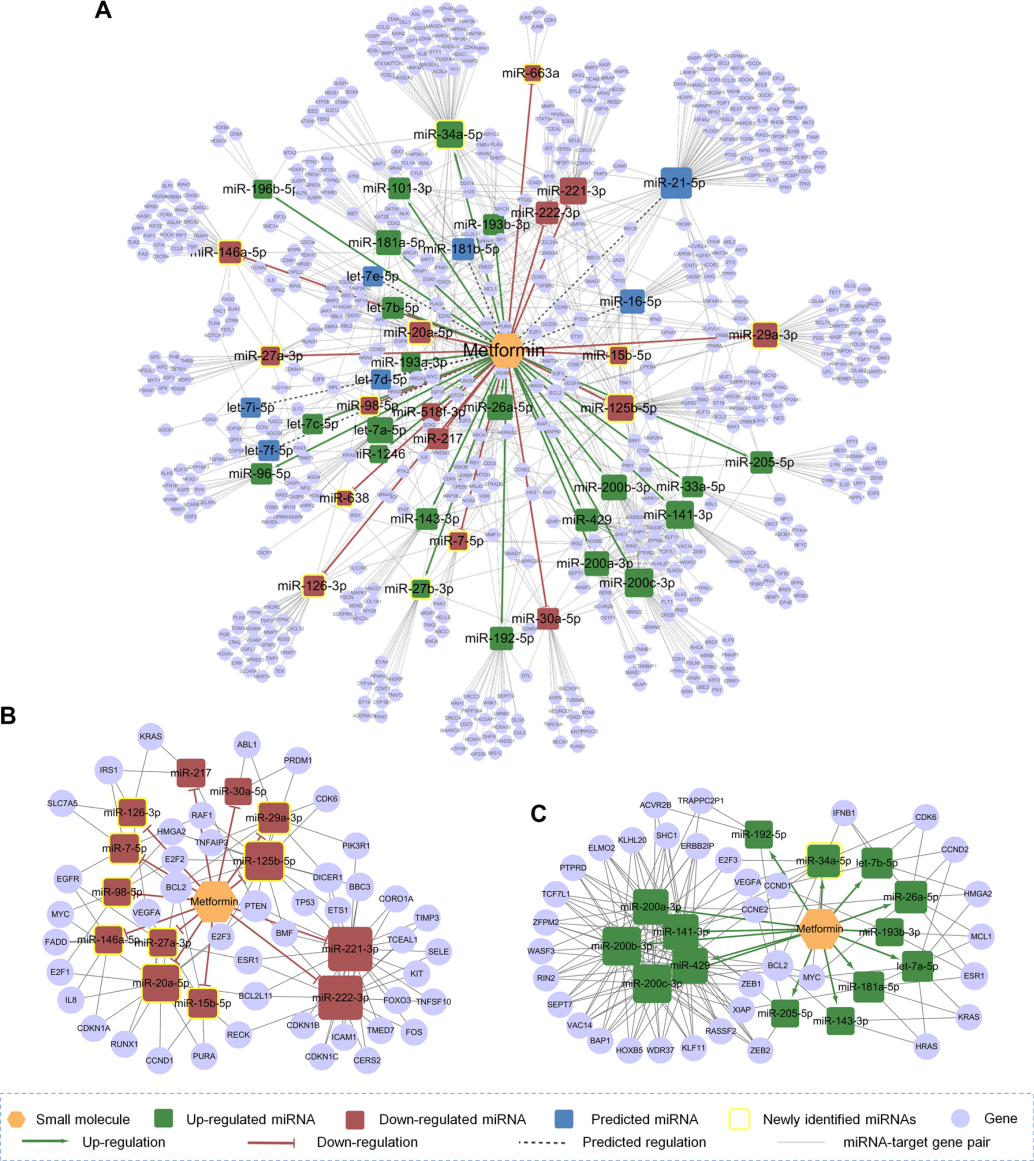
The discovered metformin-miRNA-target gene regulatory network (**A**) The whole network included 23 up-regulated miRNAs and 16 down-regulated miRNAs with their 619 target genes. (**B**) and (**C**) The down-regulation subnetwork (B) and up-regulation subnetwork (C) for metformin identified by bioinformatics analyses. Regulatory details by metformin were represented by different colors of miRNA nodes.

To further explore MOA for metformin mediated by miRNAs in breast cancer, we built a miRNA pharmacogenomic subnetwork (Figure 6A). The whole subnetwok for metformin contained 23 up-regulated miRNAs and 16 down-regulated miRNAs, by integrating literature data (Supplementary Table S4) and qRT-PCR validations (Figure 5B and 5C). Figure 6A contained 281 target genes for the 13 newly identified union miRNAs in MCF-7 or MDA-MB-231 cell lines by the qRT-PCR assays. We found that these miRNA-target genes were significantly enriched in several critical cancer-related pathways, such as cell cycle pathway (*p* = 1 × 10^−12^), ERBB signaling pathway (*p* = 2.6 × 10^−11^), and p53 signaling pathway (*p* = 3.2 × 10^−9^) (Supplementary Table S5).

The miRNA pharmacogenomic effects of anticancer drugs often involve two aspects: up-regulating the expressions of tumor suppressor miRNAs or down-regulating the expressions of oncogenic miRNAs [7]. Then we performed bioinformatics analyses to examine those two potential mechanisms. Considering the data incompleteness, miRNAs/genes with at least 2 miRNA-target gene pairs were used to build a subnetwork for examing the down-regulation by tumor suppressor miRNAs (Figure 6B), and those connected by at least 3 miRNA-target gene pairs were used to build a subnetwork for examing the up-regulation by oncogenic miRNAs (Figure 6C). Previous studies have suggested that over-expression of miR-20a-5p may mediate breast cancer by targeting multiple cancer genes, such as *PTEN* and *BCL2* [42–44]. In this study, we found that metformin down-regulated the expression of miR-20a-5p in both MCF-7 and MDA-MB-231 cell lines by integrating the SMiR-NBI model and the qRT-PCR assays (Figure 5B and 5C). Down-regulation of oncogenic miR-20a-5p and further regulating several important cancer genes (such as *PTEN* and *BCL2*) may provide a potential MOA for therapeutic effect of metformin in breast cencer (Figure 6B). A tumor suppressor miRNA, miR-34a-5p [45], targeted several critical cancer genes (Figure 6C), including *CDK6, CCND1, CCNE2, E2F3* in the cell cycle pathway and *VEGFA* in the angiogenesis pathway [35]. Here, this miRNA was up-regulated by metformin in both MCF-7 and MDA-MB-231 breast cancer cells identified by the qRT-PCR assays (Figure 5B and 5C). Altogether, down-regulation of oncogenic miRNAs (such as miR-20a-5p in Figure 6B) or up-regulation of tumor suppressor miRNAs (like miR-34a-5p in Figure 6C) by metformin may provide potential pharmacogenomic biomarkers for characterizing its anticancer effects.

## DISCUSSION

Recent studies have suggested that miRNAs play important roles in mediating responses of anticancer small molecules, as well as epigenomics and gene negative regulation at the post-transcriptional level [4]. However, systematically identifying miRNAs as pharmacogenomic biomarkers mediating drug responses is poorly explored. In this study, we built a miRNA pharmacogenomic framework, named the SMiR-NBI model, for identifying potential miRNAs as biomarkers characterizing the treatment responses of anticancer small molecules. High performance was yielded in cross-validation and the qRT-PCR assays. We showed that the SMiR-NBI model would provide a powerful approach for the identification of miRNAs as potential cancer pharmacogenomic biomarkers in the emerging research field of precision medicine. All the collected and predicted data are available on our website (http://lmmd.ecust.edu.cn/database/smir-nbi/) for future experimental validations.

Nevertheless, we should pay more attentions when using this model to predict potential miRNA pharmacogenomic biomarkers for anticancer agents. At first, the high performance of the SMiR-NBI model largely depends on data completeness and the quality of the SM-miR network. Although we constructed a high-quality dataset for the SMiR-NBI model building by removing unclear associations like “dysregulation” and conflict records, the potential data bias or false positive rates might exist in the currently public available databases. This pitfall may be solved based on the availability of more high-quality miRNA pharmacogenomic data in the near future owing to the rapid development of high-throughput technologies. Secondly, SM-miR network is a directional network with up-/down-regulation. However, the network-based inference algorithm implemented in our current SMiR-NBI model is not directional yet. Our group members are actively developing and applying directional network-based inference algorithm for SM-miR network prediction in the future. Thirdly, the current SMiR-NBI model cannot predict new miRNAs for small molecules not interlinking with the existing SMiR-NBI network, such as benzothiazole and lovastatin here [25]. Recently, our group developed a SDTNBI algorithm [46] that can predict drug targets for new chemical entities. We plan to apply our SDTNBI algorithm for predicting potential miRNAs for such kinds of novel or isolated small molecules. Finally, prediction of new miRNAs with different expression levels for small molecules with different dose-responses was lost in the current SMiR-NBI model owing to lack of the available data. Thus, development of high-quality SM-miR network with miRNA expression information and drug dose-response information is urgently needed.

In addition to anticancer potentials, the miRNA pharmacogenomic framework can be applied in several related fields, depending on the functions of miRNAs. By targeting at least 60% of all protein-coding genes, miRNAs are key regulators in the therapy strategy of cardiovascular diseases [47], neurological diseases [48], viral infection [49], *etc*. Meanwhile, miRNAs can target key metabolic enzymes or transporters mediating drug pharmacokinetics [50], adverse events and toxicity [5, 51]. In summary, we plan to develop more useful miRNA pharmacogenomic framework with a higher accuracy and robustness to identify more clinically relevant biomarkers for pharmacogenomic studies to speed up the development of precision medicine in the future.

## MATERIALS AND METHODS

### Data collection and network construction

The SM-miR network data were collected from the miREnvironment [17] and SM2miR [18] databases. Only records tested with human mature miRNAs were kept, and those analyzed by pre-miRNAs were deleted. As we only focused on small molecules, environmental factors (e.g. polypeptides) were excluded. All small molecular names were standardized by the Unified Medical Subject Headings (MeSH) [52] and the mature miRNAs were annotated by the miRBase ID [16]. To ensure the data quality, we only kept the data of miRNA expression directly regulated by small molecules supported by experiments. In addition, we checked miRNA expressions from the miREnvironment database by manual reference inspection. Associations without altered expression details were removed. The final SM-miR network was transformed into adjacent matrix after eliminating the overlap and conflicts.

The miRNA-gene targeting gene pairs were collected using the multiMiR R package [21], containing data from several resources, including miRTarBase [11], TarBase [19], and miRecords [20]. Here, only data supported by experiments tested in humans was used. Genes were standardized by GenBank Identifier [35]. Then miRNA-target gene pairs were divided into two categories based on the existing experimental evidences: (i) strong validations tested by q-PCR, western blot, reporter assay, and other low-throughput experiments, and (ii) weak validations tested by microarray, CLIP, sequencing, and other high-throughput assays [21]. If a miRNA-target gene pair was supported with both strong and weak validations, it will be labeled as strong validations. The duplicated data was removed. The final miRNA-gene network was used to illustrate the molecular mechanisms of miRNAs.

### Model building and validation

The SMiR-NBI model was built using the state-of-the-art network-based inference (NBI) algorithm as described previously [24, 25, 53–56]. Briefly, the initial resources for a given small molecule located in its regulated miRNAs. Then each miRNA averagely distributes the resources to all adjacent small molecules and the latter immediately redistribute their received resources to every neighboring miRNA. The end resource score for miRNAs stood for their likelihood to be regulated by the given small molecule [25]. Mathematically, denoting S = {s_1_, s_2_, …, s_m_} is a set of m small molecules, M = {m_1_, m_2_, …, m_n_} is a set of n miRNAs, and the initial resource matrix A can be represented as $A=\left[\begin{array}{cc} O & X\\ X^{T} & O \end{array}\right]$, where X is a m × n matrix, defined as X (i, j) = 1 if s_i_ is linked with m_j_ otherwise 0. Let $B\left(i,j\right)=\frac{A\left(i,j\right)}{\sum_{l=1}^{m+n}A\left(i,l\right)}$ the final resource matrix is F = A × B^2^, where F (i, m + j) (0 < i ≤ m, 0 < j ≤ n) is the score of s_i_ − m_j_ association. Finally, the scores for the subnetwork of a specific miRNA or small molecule were converted to standardized scores ranging from 0 to 1.

To evaluate the performance of the SMiR-NBI model, 100 times of 10-fold cross validation were performed. All links in the SM-miR network were randomly divided into ten parts of equal size, and then each part was used as test set in turn with the remaining parts as training set. To eliminate the error caused by separating data sets, all the results were yielded by a simulation of 100 independent tests. The area under the receiver operating characteristic (ROC) curve was calculated as the measure to evaluate the model performance.

### Calculating of differentially expressed miRNAs in breast cancer

We downloaded miRNA-seq data for 654 breast invasive carcinoma (BRCA) and 85 matched normal samples (12/2015) from The Cancer Genome Atlas (TCGA) [29]. The miRNA differential expression was calculated using edgeR software [57]. We then used |log_2_(fold change)| > 1 and adjusted *p*-value < 0.05 as the cutoff to definite the differentially expressed miRNAs.

### Network analyses

Network analyses were performed using Cytoscape [26]. Degrees were calculated by “Network analyzer” tool for each node in three types of SM-miR networks: global network, up-regulation network and down-regulation network, respectively. Then the top 100 small molecules or miRNAs with high degrees in global network were extracted and regulations were divided into up-regulation, or down-regulation mode. A visualization of the whole SM-miR network diagram was obtained by edge-weighted spring embedded layout, with the size of node stood for the global degrees. The heterogeneous SM-miR network was transformed into a homogeneous network with links connecting all reported small molecules characterized by the co-regulated miRNAs. MCODE plugin [28] was applied for module analyses in the homogeneous network of small molecules with parameters set at: node score cutoff 0.2, max depth from seed 5, others as default. The downstream genes targeted by miRNAs were of great importance to illustrate the miRNA functions. Degrees of genes in the miRNA-gene function subnetwork regulated by a specific small molecule were calculated to obtain the hub genes with the highest degrees, which often participated in multiple key miRNA pharmacogenomic pathways. To cut down the complexity of the regulated miRNA pharmacogenomic network, important regulatory pathways were extracted for some small molecules depending on the degrees in SM-miR network or miRNA-gene network.

### Experimental validation

Quantitative reverse transcription-PCR (qRT-PCR) assay was used to test the expression levels of the top 20 predicted miRNA candidates for metformin and the top 10 predicted potential miRNAs for tamoxifen. The human MCF-7 (hormone-positive [ER and PR] breast cancer) and MDA-MB-231 (triple negative breast cancer) cell lines used in this study were purchased from the Cell Bank of Type Culture Collection of the Chinese Academy of Sciences (Shanghai, China). The MCF-7 cell line was cultured in complete media consisting of 1640 media, while MDA-MB-231 cell line was cultured in complete media consisting of DMEM media. Both were supplemented with 10% fetal bovine serum (FBS, GIBCO, USA) and 1% antibiotic-antimycotic (ABAM Life Technologies, California, USA) under an atmosphere of 5% CO_2_ and 95% air at 37^°^C. These two breast cancer cell lines were treated for 24 hours with 1 mmol/L (mM), 5 mM and 10 mM metformin (Sigma, USA), and 100 nmol/L (nM), 500 nM and 1 μmol/L (μM) tamoxifen (Sigma, USA), respectively. Total RNA was purified using Trizol reagent (Invitrogen, CA, USA) according to the supplier's instruction. The miRNA was reversely transcribed by TransScript miRNA First-Strand cDNA Synthesis SuperMix(Transgen, China), and qRT-PCR was performed with TransScript Top Green qPCR SuperMix according to the manufacturer's protocols (Transgen, China) with an iCycler thermal cycler (Bio-Rad, USA). Relative quantification of the miRNA expression level was calculated using the comparative cycle threshold (Ct) method (2^−(ΔΔCt)^). The expression of U6 was used as the endogenous control. Reported values are the means and standard errors of results from three biological replicates. The *p* values were computed by Student's *t*-test and difference significance between groups was assessed as *p < 0.05; **p < 0.01; ***p < 0.001. The sequences of each primer used in this study were shown in Supplementary Table S6.

## Supplementary Materials Figures and Tables







## References

[1] Wheeler HE, Maitland ML, Dolan ME, Cox NJ, Ratain MJ (2013). Cancer pharmacogenomics: strategies and challenges. Nat Rev Genet.

[2] McLeod HL (2013). Cancer pharmacogenomics: early promise, but concerted effort needed. Science.

[3] Meyer UA, Zanger UM, Schwab M (2013). Omics and drug response. Annu Rev Pharmacol.

[4] Rukov JL, Shomron N (2011). MicroRNA pharmacogenomics: post-transcriptional regulation of drug response. Trends Mol Med.

[5] Koturbash I, Tolleson WH, Guo L, Yu D, Chen S, Hong H, Mattes W, Ning B (2015). MicroRNAs as pharmacogenomic biomarkers for drug efficacy and drug safety assessment. Biomark Med.

[6] Shomron N (2010). MicroRNAs and pharmacogenomics. Pharmacogenomics.

[7] Monroig PdC, Chen L, Zhang S, Calin GA (2015). Small molecule compounds targeting miRNAs for cancer therapy. Adv Drug Deliv Rev.

[8] Deiters A (2010). Small molecule modifiers of the microRNA and RNA interference pathway. AAPS J.

[9] O'Donnell KA, Wentzel EA, Zeller KI, Dang CV, Mendell JT (2005). c-Myc-regulated microRNAs modulate E2F1 expression. Nature.

[10] Velagapudi SP, Vummidi BR, Disney MD (2015). Small molecule chemical probes of microRNA function. Curr Opin Chem Biol.

[11] Hsu SD, Lin FM, Wu WY, Liang C, Huang WC, Chan WL, Tsai WT, Chen GZ, Lee CJ, Chiu CM (2011). miRTarBase: a database curates experimentally validated microRNA–target interactions. Nucleic Acids Res.

[12] Li XB, Gao L, Cui QH, Gary BD, Dyess DL, Taylor W, Shevde L, Samant RS, Dean-Colomb W, Piazza GA (2012). Sulindac inhibits tumor cell invasion by suppressing NF-κB-mediated transcription of microRNAs. Oncogene.

[13] Zhang C, Hong HX, Mendrick DL, Tang Y, Cheng FX (2015). Biomarker-based drug safety assessment in the age of systems pharmacology: from foundational to regulatory science. Biomarker Med.

[14] Banwait JK, Bastola DR (2015). Contribution of bioinformatics prediction in microRNA-based cancer therapeutics. Adv Drug Deliv Rev.

[15] Akhtar MM, Micolucci L, Islam MS, Olivieri F, Procopio AD (2016). Bioinformatic tools for microRNA dissection. Nucleic Acids Res.

[16] Kozomara A, Griffiths-Jones S (2014). miRBase: annotating high confidence microRNAs using deep sequencing data. Nucleic Acids Res.

[17] Yang QQ, Qiu CX, Yang J, Wu Q, Cui QH (2011). miREnvironment database: providing a bridge for microRNAs, environmental factors and phenotypes. Bioinformatics.

[18] Liu XY, Wang SY, Meng FL, Wang JZ, Zhang Y, Dai EY, Yu XX, Li X, Jiang W (2013). SM2miR: a database of the experimentally validated small molecules' effects on microRNA expression. Bioinformatics.

[19] Sethupathy P, Corda B, Hatzigeorgiou AG (2006). TarBase: A comprehensive database of experimentally supported animal microRNA targets. RNA.

[20] Xiao FF, Zuo ZX, Cai GS, Kang SL, Gao XL, Li TB (2009). miRecords: an integrated resource for microRNA–target interactions. Nucleic Acids Res.

[21] Ru Y, Kechris KJ, Tabakoff B, Hoffman P, Radcliffe RA, Bowler R, Mahaffey S, Rossi S, Calin GA, Bemis L (2014). The multiMiR R package and database: integration of microRNA–target interactions along with their disease and drug associations. Nucleic Acids Res.

[22] Lv YL, Wang SY, Meng FL, Yang L, Wang ZF, Wang J, Chen XW, Jiang W, Li YX, Li X (2015). Identifying novel associations between small molecules and miRNAs based on integrated molecular networks. Bioinformatics.

[23] Meng FL, Wang J, Dai EY, Yang F, Chen XW, Wang SY, Yu XX, Liu DM, Jiang W (2016). Psmir: a database of potential associations between small molecules and miRNAs. Sci Rep.

[24] Cheng FX, Liu C, Jiang J, Lu WQ, Li WH, Liu GX, Zhou WX, Huang J, Tang Y (2012). Prediction of drug-target interactions and drug repositioning via network-based inference. PLoS Comput Biol.

[25] Li J, Wu ZR, Cheng FX, Li WH, Liu GX, Tang Y (2014). Computational prediction of microRNA networks incorporating environmental toxicity and disease etiology. Sci Rep.

[26] Smoot ME, Ono K, Ruscheinski J, Wang P-L, Ideker T (2011). Cytoscape 2. 8: new features for data integration and network visualization. Bioinformatics.

[27] Seebacher J, Gavin AC (2011). Snapshot: protein-protein interaction networks. Cell.

[28] Bader GD, Hogue CW (2003). An automated method for finding molecular complexes in large protein interaction networks. BMC Bioinformatics.

[29] Cancer Genome Atlas Network (2012). Comprehensive molecular portraits of human breast tumours. Nature.

[30] Sethi S, Li Y, Sarkar FH (2013). Regulating miRNA by natural agents as a new strategy for cancer treatment. Curr Drug Targets.

[31] Masika J, Zhao Y, Hescheler J, Liang HM (2016). Modulation of miRNAs by natural agents: nature's way of dealing with cancer. RNA Dis.

[32] Cimmino A, Calin GA, Fabbri M, Iorio MV, Ferracin M, Shimizu M, Wojcik SE, Aqeilan RI, Zupo S, Dono M (2005). MiR-15 and miR-16 induce apoptosis by targeting BCL2. P Natl Acad Sci USA.

[33] Ulrich CM, Bigler J, Potter JD (2006). Non-steroidal anti-inflammatory drugs for cancer prevention: promise, perils and pharmacogenetics. Nat Rev Cancer.

[34] Mantovani A, Allavena P, Sica A, Balkwill F (2008). Cancer-related inflammation. Nature.

[35] Clark K, Karsch-Mizrachi I, Lipman DJ, Ostell J, Sayers EW (2016). GenBank. Nucleic Acids Res.

[36] Klinge CM (2015). miRNAs regulated by estrogens, tamoxifen, and endocrine disruptors and their downstream gene targets. Mol Cell Endocrinol.

[37] Mulrane L, McGee SF, Gallagher WM, O'Connor DP (2013). MiRNA dysregulation in breast cancer. Cancer Res.

[38] Isanejad A, Alizadeh AM, Shalamzari SA, Khodayari H, Khodayari S, Khori V, Khojastehnjad N (2016). MicroRNA-206, let-7a and microRNA-21 pathways involved in the anti-angiogenesis effects of the interval exercise training and hormone therapy in breast cancer. Life Sci.

[39] Tang W, Zhu JJ, Su SC, Wu W, Liu Q, Su FX, Yu FY (2012). MiR-27 as a prognostic marker for breast cancer progression and patient survival. PLoS One.

[40] Zhong SL, Li WJ, Chen ZY, Xu JJ, Zhao JH (2013). MiR-222 and miR-29a contribute to the drug-resistance of breast cancer cells. Gene.

[41] Pogribny IP, Filkowski JN, Tryndyak VP, Golubov A, Shpyleva SI, Kovalchuk O (2010). Alterations of microRNAs and their targets are associated with acquired resistance of MCF-7 breast cancer cells to cisplatin. Int J Cancer.

[42] Schwarzenbach H, Milde-Langosch K, Steinbach B, Müller V, Pantel K (2012). Diagnostic potential of PTEN-targeting miR-214 in the blood of breast cancer patients. Breast Cancer Res Tr.

[43] Zhang YQ, Zheng L, Ding Y, Li Q, Wang R, Liu TX, Sun QQ, Yang H, Peng SL, Wang W (2015). MiR-20a Induces cell radioresistance by activating the PTEN/PI3K/Akt signaling pathway in hepatocellular carcinoma. Int J Radiat Oncol.

[44] Li JY, Zhang Y, Zhang WH, Jia S, Kang Y, Zhu XY (2012). Differential distribution of miR-20a and miR-20b may underly metastatic heterogeneity of breast cancers. Asian Pac J Cancer P.

[45] Ng WL, Chen G, Wang M, Wang H, Story M, Shay JW, Zhang X, Wang J, Amin ARMR, Hu B (2014). OCT4 as a target of miR-34a stimulates p63 but inhibits p53 to promote human cell transformation. Cell Death Dis.

[46] Wu ZR, Cheng FX, Li J, Li WH, Liu GX, Tang Y (2016). SDTNBI: an integrated network and chemoinformatics tool for systematic prediction of drug–target interactions and drug repositioning. Brief Bioinform.

[47] van Rooij E, Olson EN (2012). MicroRNA therapeutics for cardiovascular disease: opportunities and obstacles. Nat Rev Drug Discov.

[48] Christensen M, Schratt GM (2009). MicroRNA involvement in developmental and functional aspects of the nervous system and in neurological diseases. Neurosci Lett.

[49] Pedersen IM, Cheng G, Wieland S, Volinia S, Croce CM, Chisari FV, David M (2007). Interferon modulation of cellular microRNAs as an antiviral mechanism. Nature.

[50] Gomez A, Ingelman-Sundberg M (2009). Epigenetic and microRNA-dependent control of cytochrome P450 expression: a gap between DNA and protein. Pharmacogenomics.

[51] Yokoi T, Nakajima M (2013). MicroRNAs as mediators of drug toxicity. Annu Rev Pharmacol.

[52] Lipscomb CE (2000). Medical subject headings (MeSH). Bull Med Libr Assoc.

[53] Cheng FX, Li WH, Wu ZR, Wang XC, Zhang C, Li J, Liu GX, Tang Y (2013). Prediction of polypharmacological profiles of drugs by the integration of chemical, side effect, and therapeutic space. J Chem Inf Model.

[54] Cheng FX, Li WH, Wang XC, Zhou YD, Wu ZR, Shen J, Tang Y (2013). Adverse drug events: database construction and in silico prediction. J Chem Inf Model.

[55] Cheng FX, Li WH, Zhou YD, Li J, Shen J, Lee PW, Tang Y (2013). Prediction of human genes and diseases targeted by xenobiotics using predictive toxicogenomic-derived models (PTDMs). Mol BioSyst.

[56] Cheng FX, Zhou YD, Li WH, Liu GX, Tang Y (2012). Prediction of chemical-protein interactions network with weighted network-based inference method. PLoS One.

[57] Robinson MD, McCarthy DJ, Smyth GK (2010). edgeR: a bioconductor package for differential expression analysis of digital gene expression data. Bioinformatics.

