# Supplementary material for: Network-based identification of microRNAs as potential pharmacogenomic biomarkers for anticancer drugs
Source: Oncotarget. 2016 Jun 14;7(29):45584–96. doi: 10.18632/oncotarget.10052 (PMC5216744; doi:10.18632/oncotarget.10052)
Supplement: Supplementary file 1 [file oncotarget-07-45584-s001.pdf]

Network-based identification of microRNAs as potential pharmacogenomic biomarkers for anticancer drugs

Supplementary Materials

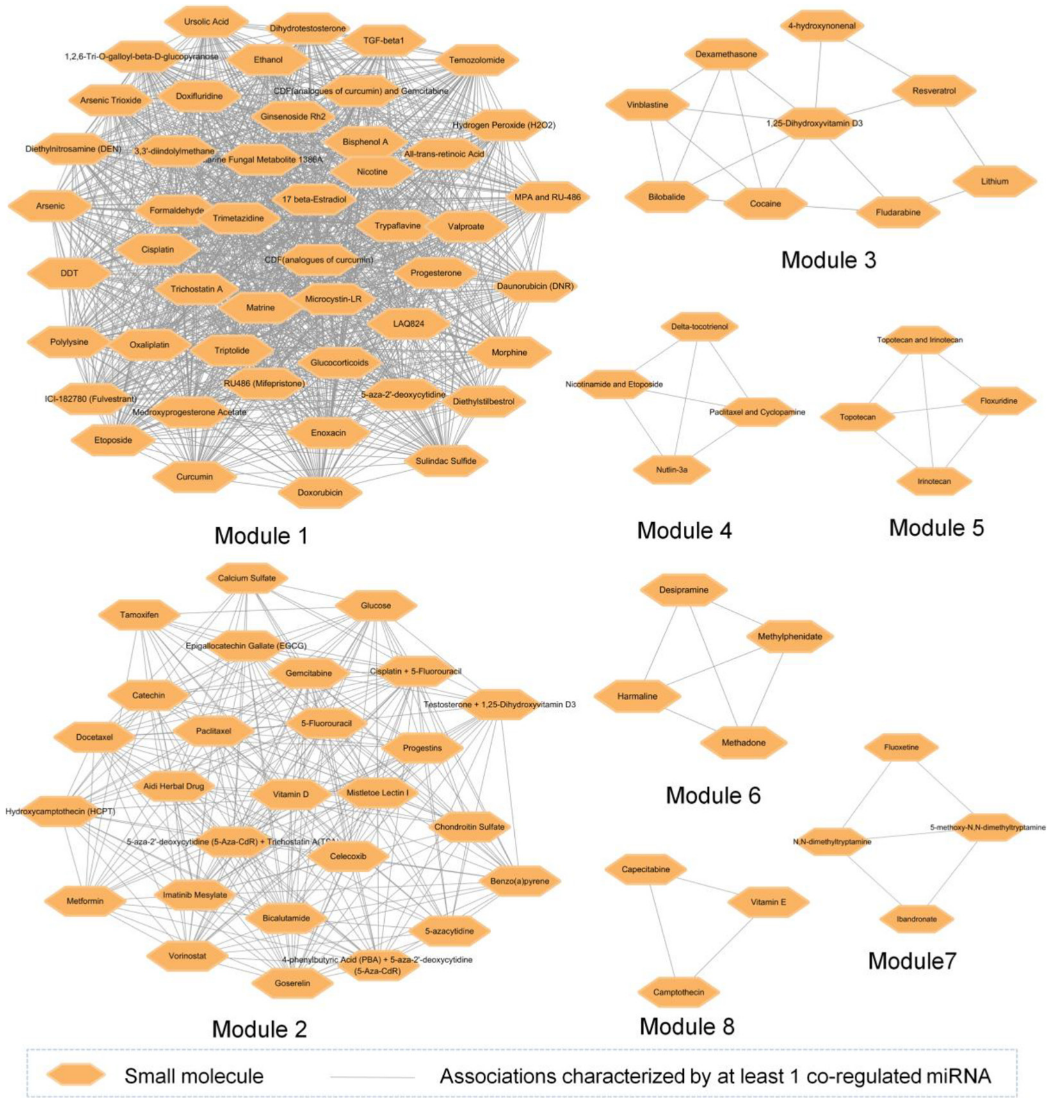

Supplementary Figure S1: All eight modules identified from the network connecting small molecules (SMs). In total, 48 small molecules were clustered in Module 1, 27 SMs in Module2, 8 SMs in Module 3, 4 SMs in Module 4~7, and 3 SMs in Module8.

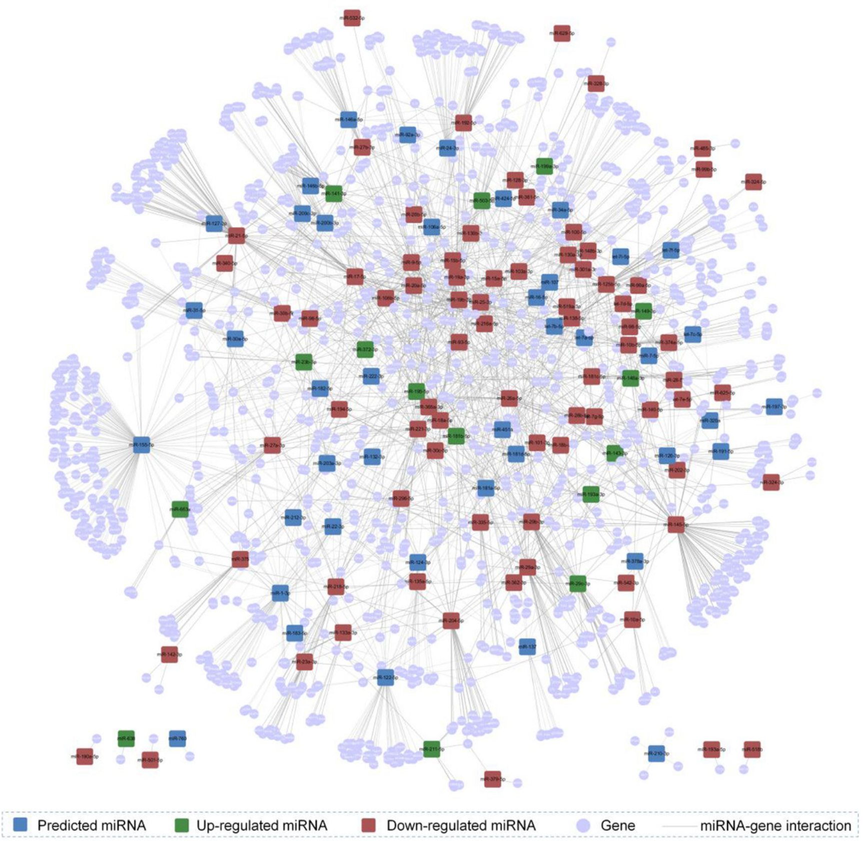

Supplementary Figure S2: The identified miRNA-target gene subnetwork for non-steroidal anti-inflammatory drugs (NSAIDs). This network included 131 previously reported miRNAs for NSAIDs (up-regulated is red versus down-regulated is green) from literatures and the top 50 predicted miRNAs (blue) for sulindac sulfide, with 10 previously reported up-regulated miRNAs (red) and the top 10 predicted miRNAs (blue) for celecoxib, identified by the SMiR-NBI model.

Supplementaty Table S1: The predicted list for the differentially expressed miRNAs in breast cancer from The Cancer Genome Atlas with 17 anti-breast cancer drugs. See Supplementary_Table_S1

Supplementary Table S2: MiRNA pharmacogenomics list for natural products, with previously reported evidences from literatures and computationally predicted miRNAs via the the SMiR-NBI model, and miRNA-target genes. See Supplementary_Table_S2

Supplementary Table S3: All validated miRNAs list directly target inflammation-related genes and their regulation details in subnetwork of non-steroidal anti-inflammatory drugs (NSAIDs)

ID	MiRNAs[a]	Inflammatory Mediator
1	miR-26b-5p miR-101-3p miR-128-3p miR-335-5p let-7b-5p miR-16-5p miR-124-3p miR-132-3p miR-137 miR-181a-5p	*PTGS2*
2	miR-211-5p miR-379-5p miR-204-5p miR-124-3p miR-1-3p	*IL11*
3	miR-98-5p let-7d-5p let-7g-5p let-7i-5p let-7f-5p	*IL13*
4	miR-26b-5p miR-15a-5p miR-15b-5p miR-29b-3p miR-16-5p miR-409-3p	*IFNG*
5	let-7e-5p miR-20a-5p miR-20b-5p miR-21-5p miR-93-5p miR-125b-5p miR-155-5p miR-92a-3p miR-337-3p	*STAT3*
6	miR-26b-5p miR-9-5p miR-15a-5p miR-21-5p let-7a-5p miR-146a-5p miR-146b-5p miR-155-5p miR-16-5p miR-92a-3p	*NFKB1*

[a]Here listed all the validated miRNAs with strong or weak validations directly targeting inflammatory mediators extracted using multiMiR R package. MiRNAs reported to be up-regulated by NSAIDs by literature were colored in green, while down-regulated ones in red. MiRNAs in the top 50 predicted lists for Sulindac Sulfide were colored in gray.

Supplementary Table S4: The newly reported miRNAs with strong validations collected by literature retrospection, compared with their ranking in the predicted list for metformin

ID	MiRNA	PMID[a]	Rank (Top)
Top 1~50 (8/50)			
1	miR-34a-5p	25576058, 24970682, 24806290, 26582729	3
2	miR-27a-3p	23803693	12
3	miR-221-3p	23648338	24
4	miR-30a-5p	25201727	25
5	miR-200a-3p	22356767	31
6	miR-205-5p	22356767	43
7	miR-141-3p	22356767	48
8	miR-222-3p	23974492, 23648338	50
Top 51~100 (4/50)			
1	miR-143-3p	25201727	61
2	miR-193a-3p	25213330	76
3	miR-193b-3p	25213330	79
4	miR-429	22356767	99
Top 101~350 (5/350)			
1	miR-217	24806290	169
2	miR-1246	23229592	185
3	miR-196b-5p	25201727	191
4	miR-33a-5p	22643892	249
5	miR-518f-3p	24806290	307
Top 351~543 (0)			
NULL			

[a]PMID showed the supported reference code in PubMed(http://www.ncbi.nlm.nih.gov/pubmed).

Supplementary Table S5: The top 20 most significantly enriched KEGG pathways for the target genes of 13 newly identified miRNAs for metformin in MCF-7 and MDA-MB-231 breast cancer cell lines via qRT-PCR assays

ID	Pathway Term	*P*-Value
1	Pathways in cancer	9.70E–31
2	Prostate cancer	1.00E–19
3	Chronic myeloid leukemia	1.90E–19
4	Pancreatic cancer	1.10E–18
5	Glioma	1.80E–16
6	Melanoma	1.90E–16
7	Bladder cancer	3.60E–15
8	Non-small cell lung cancer	4.80E–13
9	Cell cycle	1.00E–12
10	Small cell lung cancer	1.30E–12
11	Colorectal cancer	1.40E–11
12	ErbB signaling pathway	2.60E–11
13	Focal adhesion	4.10E–11
14	Endometrial cancer	5.10E–11
15	Neurotrophin signaling pathway	5.10E–11
16	p53 signaling pathway	3.20E–09
17	Renal cell carcinoma	4.90E–09
18	Progesterone-mediated oocyte maturation	9.20E–08
19	Toll-like receptor signaling pathway	1.40E–07
20	Acute myeloid leukemia	2.70E–07

Supplementary Table S6: The precursor sequences for the top 20 predicted miRNAs for metformin and top 10 predicted miRNAs for tamoxifen tested by the qRT-PCR assay

ID	miRNA	Precursor
**Metformin**		
1	miR-21-5p	TAGCTTATCAGACTGATGTTGA
2	let-7d-5p	AGAGGTAGTAGGTTGCATAGTT
3	miR-34a-5p	TGGCAGTGTCTTAGCTGGTTGT
4	miR-16-5p	TAGCAGCACGTAAATATTGGCG
5	miR-27b-3p	TTCACAGTGGCTAAGTTCTGC
6	let-7e-5p	TGAGGTAGGAGGTTGTATAGTT
7	miR-146a-5p	TGAGAACTGAATTCCATGGGTT
8	miR-15b-5p	TAGCAGCACATCATGGTTTACA
9	miR-181b-5p	AACATTCATTGCTGTCGGTGGGT
10	miR-126-3p	TCGTACCGTGAGTAATAATGCG
11	miR-125b-5p	TCCCTGAGACCCTAACTTGTGA
12	miR-27a-3p	TTCACAGTGGCTAAGTTCCGC
13	miR-98-5p	TGAGGTAGTAAGTTGTATTGTT
14	miR-638	AGGGATCGCGGGCGGGTGGCGGCCT
15	miR-20a-5p	TAAAGTGCTTATAGTGCAGGTAG
16	miR-7-5p	TGGAAGACTAGTGATTTTGTTGT
17	let-7f-5p	TGAGGTAGTAGATTGTATAGTT
18	miR-29a-3p	TAGCACCATCTGAAATCGGTTA
19	let-7i-5p	TGAGGTAGTAGTTTGTGCTGTT
20	miR-663a	AGGCGGGGCGCCGCGGGACCGC
**Tamoxifen**		
1	miR-21-5p	TAGCTTATCAGACTGATGTTGA
2	miR-27a-3p	TTCACAGTGGCTAAGTTCCGC
3	miR-27b-3p	TTCACAGTGGCTAAGTTCTGC
4	miR-29a-3p	TAGCACCATCTGAAATCGGTTA
5	miR-125b-5p	TCCCTGAGACCCTAACTTGTGA
6	miR-148a-3p	TCAGTGCACTACAGAACTTTGT
7	miR-20a-5p	TAAAGTGCTTATAGTGCAGGTAG
8	miR-16-5p	TAGCAGCACGTAAATATTGGCG
9	let-7a-5p	TGAGGTAGTAGGTTGTATAGTT
10	miR-34a-5p	TGGCAGTGTCTTAGCTGGTTGT
